# Whole-genome sequencing identified candidate genes associated with high and low litter size in Chuanzhong black goats

**DOI:** 10.3389/fvets.2024.1420164

**Published:** 2024-09-20

**Authors:** Conghui Guo, Junning Ye, Jie Liu, Zhihan Li, Ming Deng, Yongqing Guo, Guangbin Liu, Baoli Sun, Yaokun Li, Dewu Liu

**Affiliations:** ^1^Herbivore Laboratory, College of Animal Science, South China Agricultural University, Guangzhou, China; ^2^Guangdong Gene Bank of Livestock and Poultry, College of Animal Science, South China Agricultural University, Guangzhou, China; ^3^Guangdong Key Laboratory of Agricultural Animal Genomics and Molecular Breeding, South China Agricultural University, Guangzhou, China

**Keywords:** litter size, whole genome sequencing, Chuanzhong black goats, AMH, reproduction

## Abstract

The reproductive performance of goats significantly influences breeding efficiency and economic returns, with litter size serving as a comprehensive indicator. Despite this, research on the genetic control of litter size remains limited. Therefore, we aimed to explore the candidate genes affecting fecundity and compared the whole-genome sequences (WGS) of 15 high-litter (HL) and 15 low-litter (LL) size in Chuanzhong black goats. Then genetic diversity and genomic variation patterns were analyzed by phylogenetic, principal component and population genetic structure analysis, it was found that HL and LL subpopulations diverged. Population evolutionary selection elimination analysis was performed by Fst and θπ resulted in 506 genes were annotated in HL and 528 genes in LL. These genes were mainly related to Hippo signaling pathway, G protein-coupled signaling pathway, G protein-coupled receptor activity, cell surface receptor signaling pathway, gonadal and reproductive structure development. According to the significantly selected genomic regions and important pathways, we found that the g.89172108T > G variant locus in the exon of the AMH gene was significantly associated with litter size (*P* < 0.05), which could be used as an auxiliary selection gene for the high fertility of Chuanzhong black Goat.

## 1 Introduction

Goats, as highly versatile domestic animals, boast widespread distribution and adaptability globally. They exhibit commendable production capabilities, offering a diverse array of products including meat, milk, wool, and more to meet human needs. Notably, goat milk and its derivatives hold superior nutritional value compared to those of other mammals (1–3). Despite these advantages, the current domestic landscape reveals a significant shortfall in the supply of goat meat, milk, and related products, largely driven by escalating living standards. This imbalance necessitates substantial reliance on imports to satisfy domestic demand (4). Therefore, addressing the efficiency of goat production is paramount to rectify this supply-demand disparity and advance the goat industry.

Reproductive performance in goats, a critical determinant of production efficiency, is a multifaceted trait influenced by a complex interplay of genetic and environmental factors (5). Among these, litter size serves as a pivotal metric in assessing reproductive efficiency, directly impacting breeding efficacy and economic outcomes (6). The identification of fertility-related genes and genetic variations is paramount for deploying targeted breeding strategies to improve reproductive efficiency. High-throughput sequencing technology has been a powerful tool in recent years to study the relationship between traits and genes (7, 8). For example, Bi et al. found that CNV3 and CNV5 of the *BMPR1B* gene and c.71A > G loci of *PRNT* gene, especially the AA genotype, have a significant impact on the litter size, which can be used as DNA markers to participate in molecular marker-assisted selective breeding of Shaanbei white cashmere goats (9, 10). These genetic markers hold promise for molecular maker-assisted selective breeding. Despite the progress in identifying candidate genes associated with reproductive traits in goats, the discovery of dominant genes influencing litter size, akin to the *FecB* gene in sheep, remains elusive (11). This gap highlights the necessity for further comprehensive research to uncover such genes in goats, which would significantly contribute to the refinement of breeding programs.

The Chuanzhong black goat, a breed recently introduced to South China, demonstrates rapid growth, superior meat production, and broad adaptability (12). However, its reproductive performance displays considerable variability, with some individuals achieving a lambing rate and kidding rate exceeding 2.5, while some goats fall below 2.0. This variability presents both a challenge and an opportunity for genetic improvement. Accordingly, this study endeavors to explore candidate genes related to reproductive performance and their regulatory networks. We conducted WGS to investigate the population genetics of high and low-litter size within the Chuanzhong black goat and endeavored to identify genomic regions and genes associated with reproduction. Our findings are intended to provide a scientific foundation for the selection and improvement of this breed, thereby contributing to the theoretical and practical optimization of the goat breeding industry in South China and potentially informing global breeding practices.

## 2 Materials and methods

### 2.1 Sample collection and whole genome sequencing

At Wens Liangdong Goat Farm, we conducted a screening process on 30 black goats with homogeneous genetic backgrounds, robust physical conditions, comparable ages, uniform breeding environments, and identical parity, yet varying litter sizes. This selection comprised 15 high-litter (HL) goats, exhibiting an average litter size exceeding 2.6 in the 2nd, 3rd, and 4th births, alongside 15 low-litter (LL) goats, manifesting an average litter size below 1.7. After the DNA extraction of the selected goat ear samples using the TIANamp Genomic DNA Kit (TIANGEN, CHINA), their purity and integrity were evaluated. Subsequently, the qualified DNA samples underwent library construction and sequencing at Novogene Co., Ltd. (13). The sequencing libraries were prepared with the NEB Next^®^ Ultra™ DNA Library Prep Kit for Illumina (NEB, USA), following the manufacturer's protocols, with index codes assigned to each sample. Index-coded samples were clustered using the Illumina PE Cluster Kit (Illumina, USA) on a cBot Cluster Generation System. Following cluster generation, sequencing of the DNA libraries was conducted on the Illumina platform, resulting in the generation of 150 bp paired-end reads (14).

### 2.2 Data quality control, mapping, and annotation

Before proceeding with the analysis of sequencing data, it is imperative to conduct quality control measures to ensure the precision and reliability of the data. This involves filtering the raw data to ensure that the sequencing error rate at each base position is maintained below 1%. Then valid sequencing data was mapped to the reference genome (https://ftp.ncbi.nlm.nih.gov/genomes/all/GCF/001/704/415/GCF_001704415.1_ARS1) by Burrows-Wheeler Aligner (BWA) software (Version: 0.7.8) with parameter as mem -t 4 -k 32 -M to get the original mapping results (15).

The variation detection in this study primarily focused on single nucleotide polymorphism sites (SNPs), which are primarily focused on SAMtools (version 1.2) with the parameters as Dp4-miss0.1-maf0.05 (16). To reduce the error rate of SNP detection, The depth of the variate position < 4 and mapping quality (MQ) < 20 were filtered out, and the filtered high-quality SNPs were functionally annotated with ANNOVAR software (Version: 2013-05-20) (17).

### 2.3 Population structure

Population genetic structure can reflect the relationships and evolutionary process between groups or within populations. Population structure analysis was performed using PLINK 1.9 (18). The input file-Ped file of PLINK 1.9 was first created to describe individual genotype data and kinship, then use the ADMIXTURE V 1.3.0 software (19) to construct the population genetic structure and population lineage information. In this study, three methods of population genetic structure analysis were used: phylogenetic tree, PCA, and Structure (20).

### 2.4 Population evolution selection elimination analysis

According to the filtered SNPs, population genome scanning of HL and LL groups of Chuanzhong black goats were performed using PopGenome software (21) with sliding window algorithm. The size of the selection and elimination window was determined according to the density of SNPs at the genome level. The calculation of windows with different lengths showed that starting from 100 kb, the number of SNPs in the window began to stabilize, so a 100 kb window was chosen for selection elimination analysis. Based on the Fixation indices (F-statistics) (22) of population differentiation, Fst values were calculated for each window using vcftools_v0.1.14 software (23) with a sliding window of 100 kb interval and 50% overlap as a step to assess the population genetic structure among subpopulations. Based on nucleotide diversity (θπ) (24), it involves sliding a window of a certain θπ size across the genome and analyzing the differences in population genetic information (SNPs) within the sliding window. In this study, vcftools_v0.1.14 was used with 100 kb intervals as sliding windows, 50% overlap as step size, to compute the population θπ value. We identified and retrieved genes annotated within the overlapping regions representing the top 5% selections by both θπ and Fst (25).

### 2.5 Gene function enrichment

For gene functional enrichment analysis, we selected the top 5% regions identified by θπ and the top 5% regions identified by Fst, then we extracted overlapping regions and annotated the genes within those regions. All candidate genes were first mapped to each term in the GO database, the number of target genes mapped to each term was calculated, then the GO entries that were significantly enriched in the candidate target genes were identified using the hypergeometric test. The major biological functions of the candidate target genes can be determined by GO functional significance enrichment analysis (26). KEGG pathway analysis was performed on candidate genes using the KOBAS website and pathways with corrected *p*-value ≤ 0.05 were selected as significantly enriched in the candidate genes (27). The pathway significant enrichment analysis can identify the most important biochemical metabolic pathways and signal transduction pathways in which the candidate genes are involved.

### 2.6 Re-sample collection and AMH gene polymorphism detection

*AMH* is the highly prioritized gene within the HL group. To authenticate the selected SNPs, we procured an additional 161 ear samples from healthy female Chuanzhong black goats at Guangdong Wen's Liangdong Goat Farm. These samples were sourced from animals with documented litter sizes for their first three births, and devoid of any instances of abortion, dystocia, or stillbirths. Subsequently, ear DNA extraction was performed. Next, specific primers were designed for two SNP sites on the *AMH* gene exon (g.89172108T > G F: AGCGAAGGTGGTCAAGTCAC g.89172183G > A R: AGGAGATAGGGACTGCCCTG). PCR amplification was performed using these primers, and the qualified PCR products were sent to BGI Genomics Co., Ltd. for Sanger sequencing. Then, use SeqMan Pro (https://www.dnastar.com/software/lasergene/seqman-ultra/) to examine the sequencing chromatograms and determine the genotypes of the 2 SNP mutation sites in each goat sample. Then, the sequencing results were compared with the gene fragments to find out the SNP variation sites, and the gene frequency was analyzed using SPSS 25 (https://www.ibm.com/support/pages/downloading-ibm-spss-statistics-25) to determine the impact of the mutation sites on the litter size.

## 3 Results

### 3.1 Gene sequencing and general features of samples

**Figure 1:**
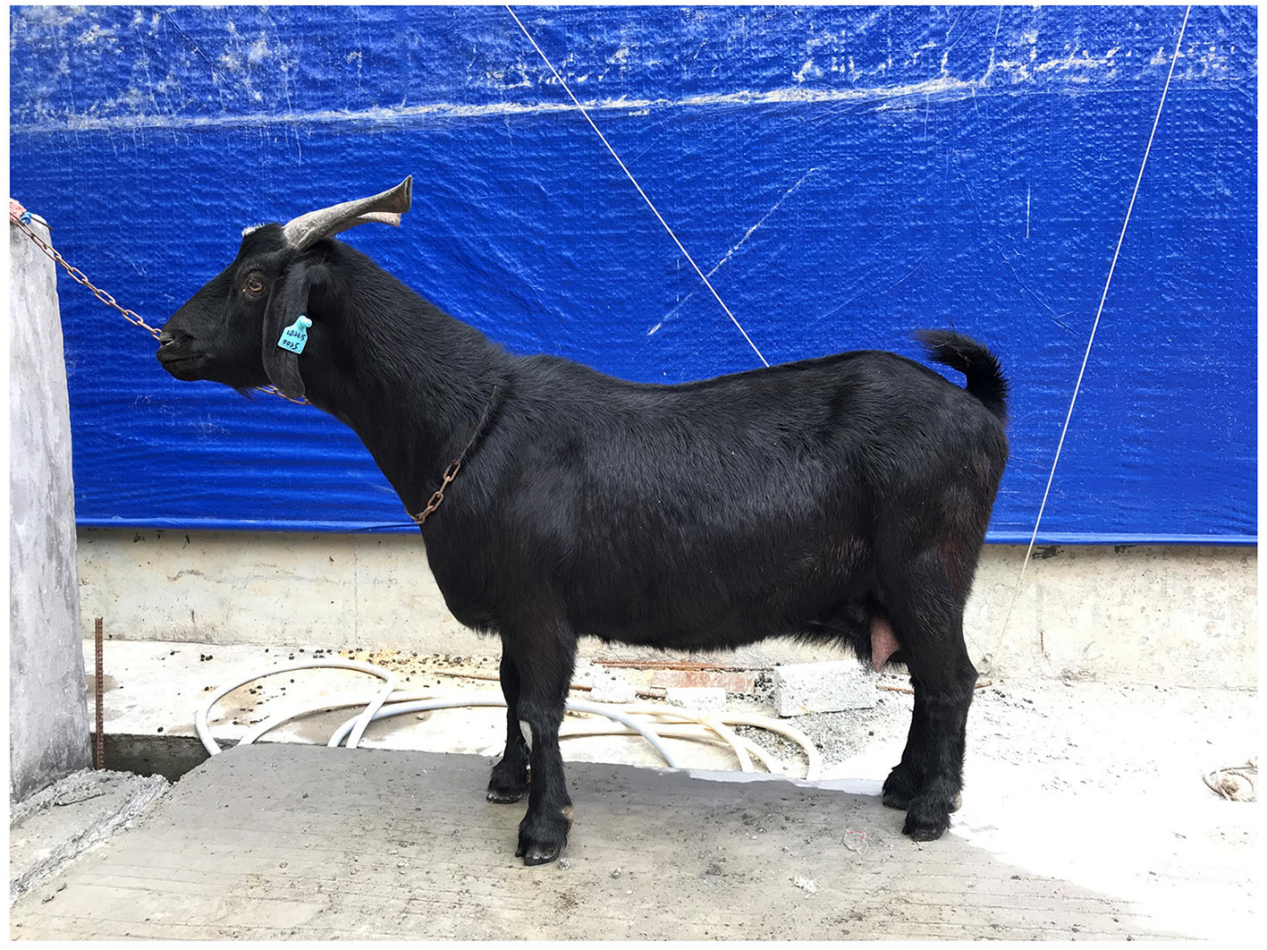
Chuanzhong black goat.

Per the local standard DB51T 2355-2017 of Sichuan Province, the reproductive performance of female Chuanzhong black goats (Figure 1) is categorized into four classes (Table 1). Specifically, a litter size exceeding 2.5 is designated as super class, over 2.4 as first class, over 2.2 as second class, and over 2.0 as third class. This study focuses on extreme populations with litter size above super class (≥2.6) or below third class ( ≤ 1.7). WGS was performed on 15 HL and 15 LL Chuanzhong black goats using Illumina platform. We obtained 851.26GB (Q20 ≥ 96.74%, Q30 ≥ 91.53%) clean data with 43.03% average GC content. As show in Supplementary Table S1. Sequencing depth and coverage statistics are shown in Supplementary Table S2.

**Table 1:** Reproductive performance classification of female Chuanzhong black goats.

**Class**	**Super**	**First**	**Second**	**Third**
Litter size	≥2.5	≥2.4	≥2.2	≥2.0

A total of 9,890,068 SNPs were obtained after the data filtered by SAMTOOLS software. Among them, 44,408 (0.45%) were in the 1 Kb region upstream of the gene and 54,284 (0.55%) were in the 1 Kb region downstream of the gene. There were 61,544 exon SNPs (0.62%), most of them were synonymous mutations (0.37%) and non-synonymous mutations (0.25%); a small part was acquired stop codon mutations (0.002 %), and loss of stop codon variation (0.25%). In addition, there were 132 (0.0013%) SNPs located in the splice site. The SNP mutation types of goats were mainly conversions of T/C and G/A (Supplementary Figure S1B). The software annotation revealed that the number of chromosomal SNPs in the HL and LL groups showed an overall decreasing trend starting from chromosome 1 (Supplementary Figure S1A), with the highest number of chromosome 1 SNPs and the lowest number of chromosome 25 SNPs.

### 3.2 Population structure and characterization of HL and LL Chuanzhong black goats

**Figure 2:**
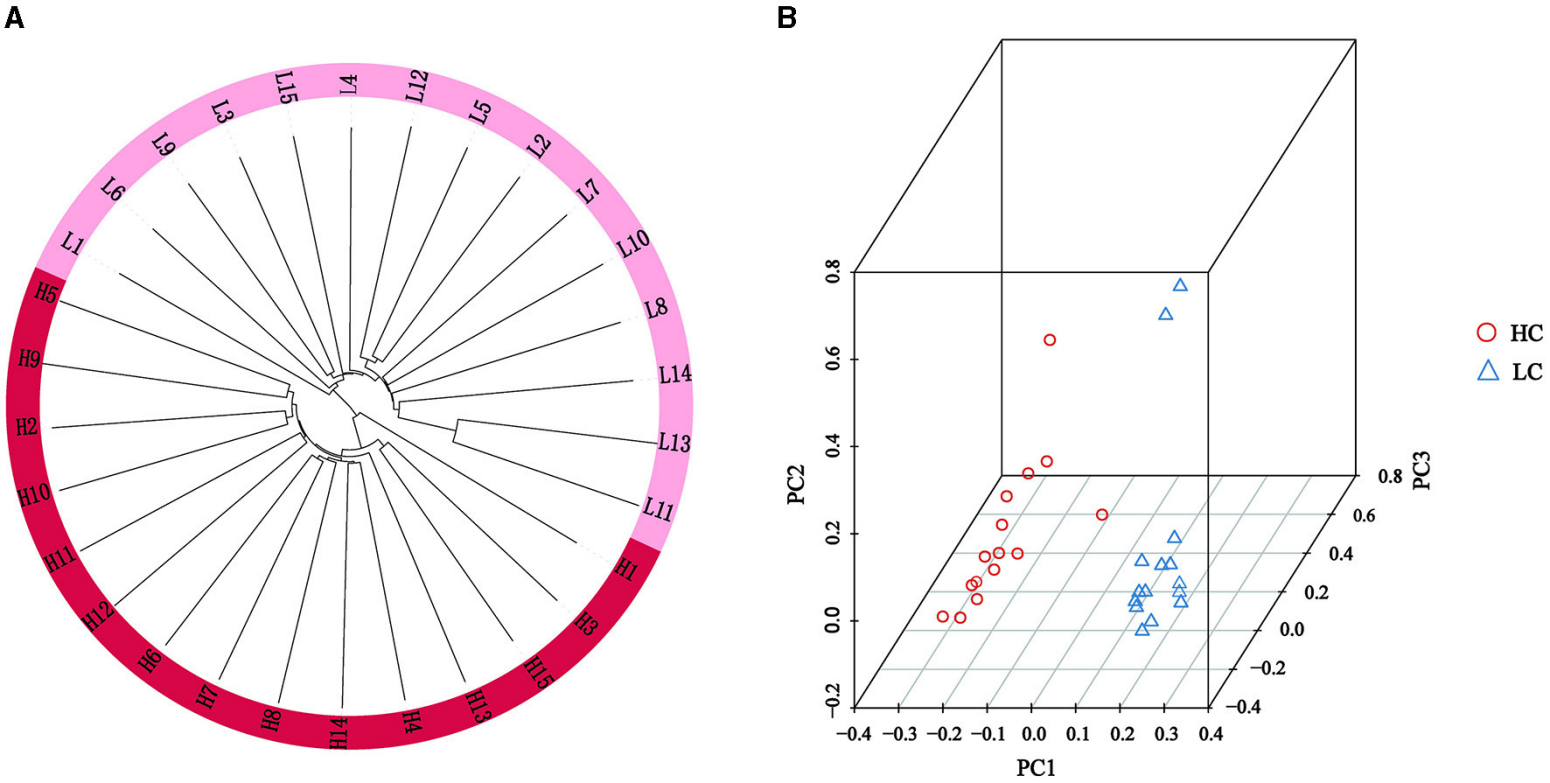
Population structure and characterization of high and low litter sizes in Chuanzhong black goat. **(A)** Phylogenetic tree of HL and LL groups; **(B)** PCA of HL and LL groups.

To investigate the relationship among experimental individuals, phylogenetic tree and PCA were conducted on all individuals. The results of the phylogenetic tree constructed by Treebest showed that although the two groups of high and low litters came from the similar genetic background, there was obvious genetic differentiation between the two groups (Figure 2A). The PCA plot also demonstrated clear separation between the HL and LL groups, classifying them into two distinct populations (Figure 2B).

### 3.3 Mining the genetic of high and low litter size

**Figure 3:**
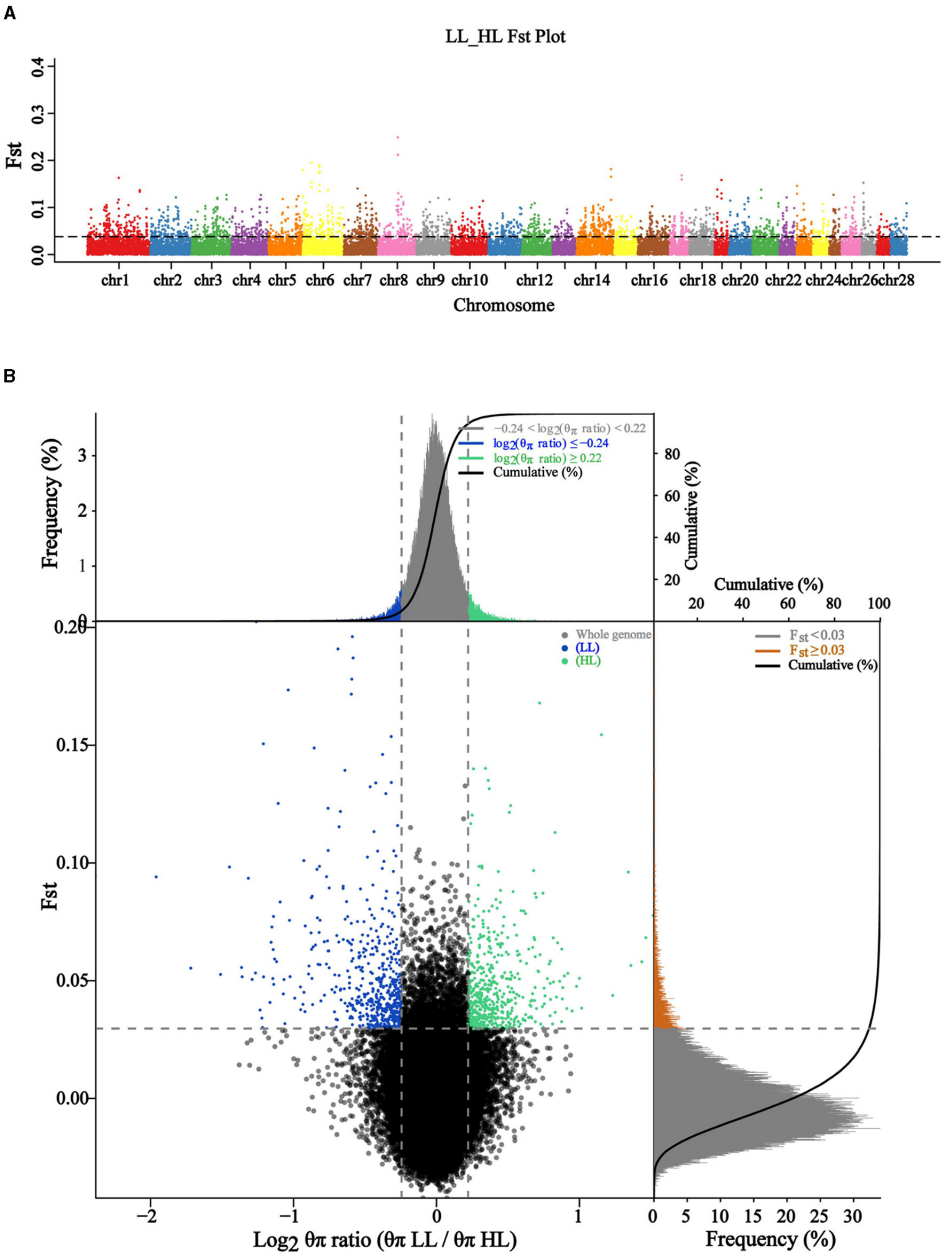
Genetic excavating on high and low litter size in Chuanzhong black goat. **(A)** Distribution of autosomal Fst in HL and LL groups; **(B)** Fst & θπ selection elimination analysis for HL and LL groups.

To identify genetic variants or loci associated with the differences in reproductive performance between HL and LL groups, we performed a population evolutionary selection elimination analysis to calculate the Fst values between two experimental groups, a total of 59,067 windows were obtained (Figure 3A). We conducted the Fst & θπ joint analysis by scanning the genomes of the HL and LL groups, which aimed to screen out stronger candidate genes related to the target traits, this involved selecting windows that simultaneously reached the top 5% for both Fst values and θπ values. The results of selected regions were shown in Figure 3B. It can be observed that the green region represents the top 5% selected regions in the HL group [log2 θπ ratio (θπLL/θπHL) < −0.22, Fst > 0.03], with annotations for a total of 506 genes. Among the genes annotated by SNPs with maximum Fst values were *SKAP1, CBX1*, and *SNX11*, in addition to *AMH, HESX1, WNT2, PRDM1*, and *TAF4*. The blue region represents the top 5% selected regions in the LL group [log2 θπ ratio (θπLL/θπHL) < −0.24, Fst > 0.03], with annotations for a total of 528 genes. Include *ARRDC1, GMDS, MRPL40, RAPSN*, and *SLC39A13*. In addition, except for the selected genes specific to the HL and LL groups, there were 8 common selected genes in the selected regions of both groups, namely *CEP350, EYA1, GMDS, KIAA2012, MTCL1, NRG3, PRDM1*, and RABGAP1L.

**Figure 4:**
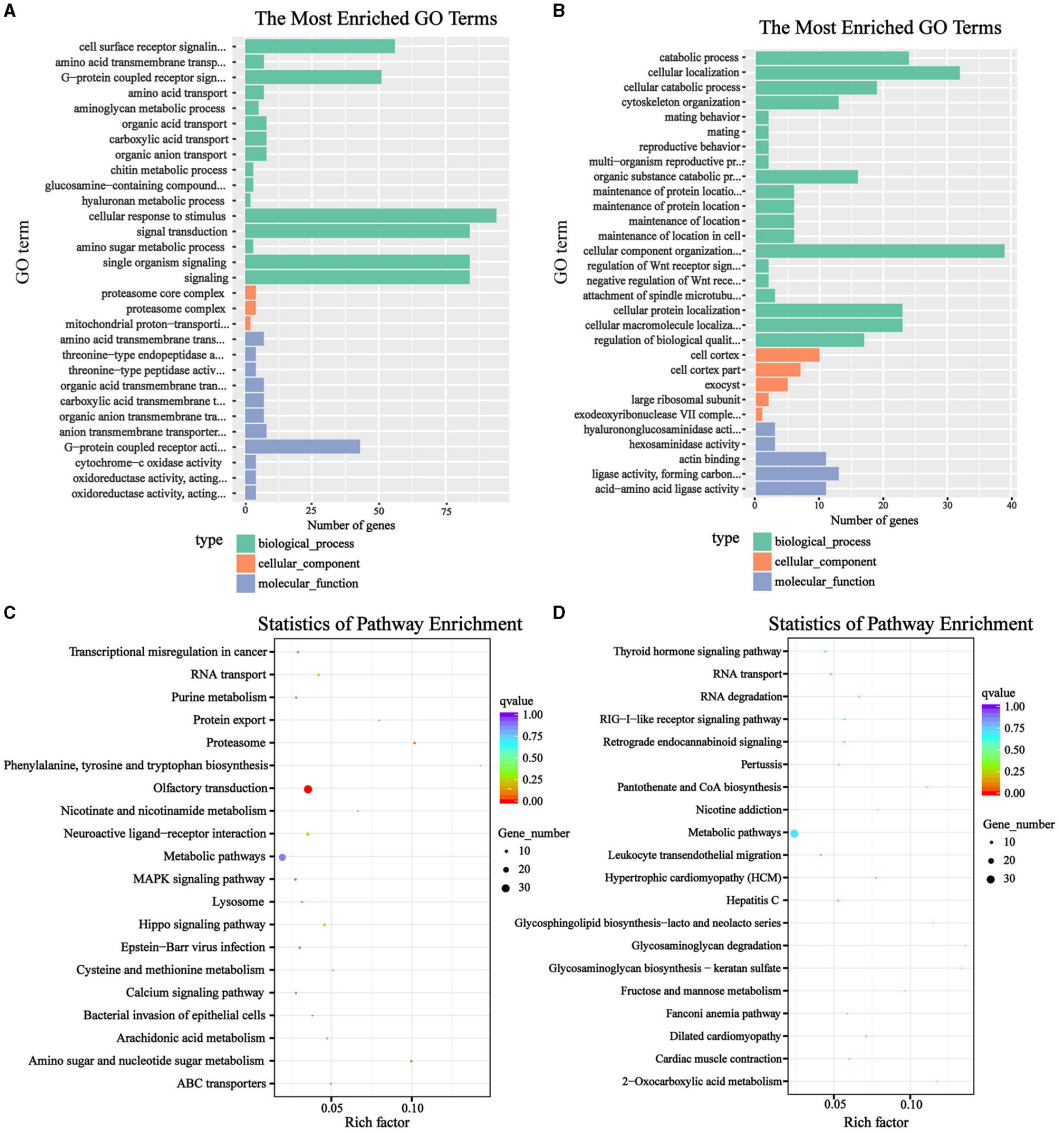
Functional enrichment of genes related to litter size. **(A, B)** GO enrichment analysis of HL and LL group; **(C, D)** KEGG enrichment analysis of HL and LL group.

Next, the GO and KEGG functional enrichment were performed on the strongly selected genes obtained by the Fst & θπ analysis in the HL and LL group, Terms significantly enriched among the candidate genes were selected based on an overrepresented *p-*value threshold of ≤ 0.05. In the HL group, a total of 121 GO function items were significantly enriched, mainly including cell surface receptor signaling pathway, G-protein coupled receptor activity, cellular response to stimulus, signal transduction, single organism signaling, reproductive system development, ATPase activity, coupled to transmembrane movement of ions, rotational mechanism, etc. (Figure 4A). There were 7 KEGG items significantly enriched, namely olfactory transduction, proteasome, amino sugar and nucleotide sugar metabolism, hippo signaling pathway, neuroactive ligand-receptor interaction, RNA transport and arachidonic acid metabolism (Figure 4C). This showed that the HL group had a more active biological response, a more sensitive sense of smell, etc. While in the LL group, 132 GO items were significantly enriched, including cellular catabolic process, cytoskeleton organization, negative regulation of WNT receptor signaling pathway, negative regulation of signal transduction, ligase activity, etc. (Figure 4B). And 14 KEGG items were significantly enriched. They were hypertrophic cardiomyopathy, dilated cardiomyopathy, glycosaminoglycan degradation, hepatitis C, RNA degradation, retrograde endocannabinoid signaling, RIG-I-like receptor signaling pathway, etc. (Figure 4D). Apparently, more disease-related pathways were enriched in the LL group, which was very detrimental to reproductive ability.

### 3.4 Association analysis of AMH gene polymorphism and litter size performance

The analysis of Fst and θπ revealed a highly selected gene, AMH, within the HL group, hypothesized to exert an influence on goat litter size in conjunction with existing literature. Therefore, specific primers were designed targeting two SNP mutation sites in the AMH gene exon. PCR amplification was performed using DNA templates from 161 Chuanzhong black goats (Supplementary Table S3). The results revealed polymorphisms at both SNP mutation sites in the AMH gene exon, and the base types of the mutation sites were consistent with the results of whole-genome resequencing. Among them, g.89172108T > G exhibited moderate polymorphism (0.25 < PIC < 0.5), while g.89172183G > A showed low polymorphism (PIC < 0.25). Chi-square tests showed that the *P-*values for g.89172108T > G and g.89172183G > A were both >0.05, indicating that they were in Hardy-Weinberg equilibrium.

Subsequently, SPSS 25 was used to analyze the association between different genotypes of AMH gene and different litters of Chuanzhong black goat and the average number of lambs produced in the first three litters. As shown in Table 2, the AMH g.89172108T > G variant locus was significantly (*P* < 0.05) associated with the average number of lambs produced in the first three litters, and the TT genotype was significantly higher than the GG genotype, and it was also observed that the number of lambs produced by the TT genotype was higher than that of the TG and GG genotypes in the first, second and third litter. However, the g.89172108T > G variant site showed no significant association with the overall litter size of Chuanzhong black goats (*P* > 0.05).

**Table 2:** Association analysis between SNP variant site of AMH and litter size in Chuanzhong black goat.

**Variant site**	**Genotype**	**Firstborn**	**Second litter**	**Third litter**	**Average litter size**
g.89172108 T>G	TT (84)	2.11 ± 0.76	2.33 ± 0.84	2.44 ± 0.7	2.3 ± 0.53^a^
	TG (59)	1.81 ± 0.68	2.17 ± 0.72	2.07 ± 0.85	2.02 ± 0.45^ab^
	GG (18)	1.76 ± 0.61	2.11 ± 0.76	2.08 ± 0.7	1.98 ± 0.48^b^
g.89172183 G>A	GG (129)	1.66 ± 0.6	2.03 ± 0.69	2.06 ± 0.84	1.92 ± 0.46
	GA (32)	1.86 ± 0.67	2.19 ± 0.77	2.13 ± 0.74	2.06 ± 0.48

Data in the same column with different letters on the shoulder indicate significant differences (*P* < 0.05), and data with the same letter or no letter on the shoulder indicate non-significant differences (*P* > 0.05).

## 4 Discussion

Optimizing litter size is crucial for enhancing livestock productivity and farm profitability, as it directly correlates with reproductive efficiency. To better understand the genetic underpinnings of high fecundity, this study employed WGS on 15 high and 15 low litter size Chuanzhong black goats. The goal was to elucidate gene expression profiles linked to reproductive performance and identify potential marker genes for selective breeding.

The selected sample consisted of 30 goats from the same genetic background and farm, ensuring uniformity in environmental conditions. Population structure analysis and principal component analysis both revealed significant genetic differentiation between the high and low litter size groups, underscoring the distinct genetic bases of reproductive traits. These findings are consistent with prior research by Kang et al. (28), who reported global differences in DNA methylation between high and low litter size goat ovaries. Similarly, Zhang et al. (29) identified significant genomic copy number variations (CNVs) associated with litter size differences, further supporting the notion of genetic divergence in reproductive traits. The current study builds on these findings by highlighting differences in gene expression, not only within goats (30) but also in other species like pigs (31) and mice (32). Interestingly, the observed genetic convergence in prolificacy between goats and sheep (33) suggests that similar genetic trends may govern reproductive traits across different species.

Goat reproductive traits are governed by polygenic inheritance and are influenced by a myriad of internal and external factors, making them complex quantitative traits. These traits involve various physiological processes such as ovarian follicle development, oocyte maturation, and embryo development. Understanding these traits has both theoretical and practical implications for animal breeding. However, the heritability of these traits is relatively low, and environmental factors often slow breeding progress (34). Selective elimination remains a pivotal tool in livestock management, as it enhances productivity, health, and economic viability by culling undesirable traits or genotypes. This study's selective elimination analysis identified candidate genes and variant sites related to reproduction, which could improve reproductive performance and enhance production efficiency. Through combined Fst and θπ analysis, a total of 506 genes were selected in the high litter size group. Notably, the genes SKAP1, CBX1, and SNX11 were among those annotated with the SNPs having the highest Fst values. SKAP1, an immune cell adapter, plays a role in T cell immune responses (35), which are vital for ovarian function (36). CBX1, a member of the heterochromatin protein family, is involved in chromatin condensation and transcriptional regulation, processes that significantly influence both sperm development and cognitive function (37, 38).

In addition, we found *AMH* related to reproduction in the genes selected and enriched signaling pathways by the HL group. *AMH* belongs to the transforming growth factor-beta (TGF-β) superfamily, which is produced by granulosa cells of early-growing follicles in the ovary and is associated with the antral follicle number. It is most strongly expressed in the granulosa cells of preantral and small antral follicles, whereas its expression decreases after dominant follicle selection and is absent in atretic follicles (39). This dynamic pattern has been confirmed in various animals, including rats (40), humans (41), cattle (42), sheep (43), and goats (44), suggesting that *AMH* is a major regulator of early follicular growth. Furthermore, Han et al. (45) found a significant association between a non-synonymous SNP (g.89172108A > C) in the *AMH* and litter size in the second parity of Dazu black goats (46). In this study, we discovered two non-synonymous SNP variants in the *AMH* of Chuanzhong Black goats: g.89172108T > G and g.89172183G > A. It was validated that the g.89172108T > G variant was significantly associated with the average litter size of the first three parities (*P* < 0.05). The TT genotype showed significantly higher litter size than the GG genotype, and across the data of the first, second and third litter size, the TT genotype was higher than that of the TG and GG genotypes. It is speculated that Chuanzhong black goats with the TT genotype may possess more ovarian reserves, leading to an increase in litter size. This genotype could be a potential molecular marker for selecting individuals with high reproductive performance in goats.

The functional enrichment of selected genes provides valuable insights for identifying candidate genes related to reproductive traits. Several significantly enriched pathways closely associated with reproduction were identified, including the Hippo signaling pathway in the KEGG database and G-protein coupled receptor signaling, G-protein coupled receptor activity, cell surface receptor signaling, gonad development, reproductive organ development, and reproductive system development in the GO database. The Hippo signaling pathway is an evolutionarily conserved pathway that regulates organ size by controlling cell proliferation, differentiation, apoptosis, and cell fate determination (47). The Hippo pathway and its effector, *YAP1*, are crucial for fine-tuning ovarian physiology, including cell fate determination, follicle activation, growth and differentiation, and steroidogenesis (48, 49). Dysregulation of this pathway can lead to excessive activation of *YAP1* in granulosa cells, resulting in the disruption of tissue homeostasis and decreased fertility. G protein-coupled receptors (GPCRs) are the largest transmembrane receptor superfamily found in the genomes of humans, mice, nematodes, fruit flies, and other organisms. They play crucial roles in regulating various physiological functions, including external and internal secretion, smooth muscle and cardiac contraction, fluid homeostasis, and immune responses (50, 51). Research has revealed that GPCRs can activate or inhibit the Hippo-YAP pathway, with the Hippo pathway established as a critical signaling branch downstream of GPCRs (52). This indicates that the selected genes of the high and low litter size groups of Chuanzhong black goats in this study may be related to the reproductive process by participating in the regulation of Hippo signaling pathway, G protein-coupled receptor activity, G protein-coupled signaling pathway and reproductive system development. Future research should focus on validating these findings in other goat breeds and livestock species, as well as exploring the environmental interactions with these genetic markers to optimize breeding strategies.

## 5 Conclusion

Our study showed that based on WGS data, there was a divergence between the high and low litter size subpopulations of Chuanzhong black goats. The evolutionary selection for high-yield traits may implicate pathways such as the Hippo signaling pathway, G protein-coupled signaling pathway, G protein-coupled receptor activity, cell surface receptor signaling pathway, gonadal development, reproductive structure development. Furthermore, we identified a significant association between the mutation locus g.89172108T > G in the exon of the AMH gene and the average number of lambs within the first three litters (*P* < 0.05), suggesting its potential utility as a molecular marker for assisted selection.

## Data Availability

The datasets presented in this study can be found in online repositories. The names of the repository/repositories and accession number(s) can be found in the article/Supplementary material.
