# Supplementary material for: Whole-genome sequencing identified candidate genes associated with high and low litter size in Chuanzhong black goats
Source: Front Vet Sci. 2024 Sep 20;11:1420164. doi: 10.3389/fvets.2024.1420164 (PMC11449896; doi:10.3389/fvets.2024.1420164)
Supplement: Supplementary file 2 [file Data_Sheet_2.PDF]

ICS 65.020.30
B43

DB51

四 川 省 地 方 标 准

DB 51/ XXXXX—2017

川中黑山羊

（报批稿）

2017－XX－XX 发布 2017－XX－XX 实施

四川省质量技术监督局 发布

目　次

前言 II
1　范围 1
2　品种特性 1
3　等级评定 2

前　言

本标准依据GB/T 1.1－2009给出的规定进行编写。

本标准由四川省农业厅提出并归口。

本标准由四川省质量技术监督局批准。

本标准起草单位：四川省畜牧总站、成都市动物疫病预防控制中心、资阳市农业局、金堂县农林局、乐至县农业局、成都蜀新黑山羊产业发展有限责任公司、乐至县天龙农牧科技有限公司起草。

本标准起草人：周光明、王小强、杨舒慧、周立新、刘金鑫、王良修、古维刚、卿静、黄长清、肖静容、林世武、付锡山、陈艳。

川中黑山羊

1　范围

本标准规定了川中黑山羊的品种特性和等级评定。
本标准适用于川中黑山羊品种鉴定和种羊等级评定。

2　品种特性

2.1　原产地及分布

川中黑山羊分为金堂和乐至两个类型,原产地为金堂县、乐至县，成都市的青白江区、德阳市的中江县等相邻县（区）有一定数量分布。该品种具有生长发育快、繁殖力高、产肉性能好、适应范围广等特点。

2.2　外貌特征

2.2.1　被毛

全身被毛黑色，毛短富有光泽，被毛内层着身绒毛。乐至型少数头顶部有“栀子花”样白毛。

2.2.2　体型外貌

体质结实，结构匀称，体型高大。头大小适中，有角或无角，公羊角粗大，向后弯曲并向两侧扭转，母羊角较小，呈外八字型。耳有垂耳、半垂耳和立耳。公羊鼻梁微拱，母羊鼻梁平直。成年公羊颌下有毛髯，少数母羊颌下有毛髯。少数羊颌下有肉髯。颈长短适中，前胸深广，肋骨开张，背腰平直，四肢粗壮，肢势端正，蹄质结实。公羊体态雄壮、前躯发达，睾丸发育良好，雄性特征明显；母羊体态清秀，后躯发达，肌肉丰满，乳房发育良好。

2.3　生产性能

2.3.1　体重

公羊、母羊六月龄体重分别为30kg～31 kg、 26 kg～27 kg，公羊、母羊周岁体重分别为41 kg～42kg、35 kg～36 kg，公羊、母羊成年体重分别为65 kg～68kg、48 kg～50kg。

2.3.2　产肉性能

12月龄公羊胴体重达20kg以上,屠宰率49%以上，净肉率37 %以上；母羊胴体重达18kg以上,屠宰率47%以上，净肉率35%以上。

2.3.3　繁殖性能

母羊的初情期5月龄～6月龄，公羊性成熟期6月龄～7月龄。初配年龄公羊10月龄～12月龄，母羊8月龄～10月龄。母羊常年发情，发情周期20d±2d，发情持续期48h±6h，妊娠期149d±3d，产羔率：初产190%，经产240%。

3 等级评定

3.1 体型外貌等级划分

体型外貌评分见表1。

表1 体型外貌评分表

项 目	评 分 要 求	评 分	
		公羊	母 羊
整体结构	体质结实，结构匀称。头大小适中，有角或无角。鼻梁微拱。耳为垂耳或半垂耳或立耳。成年公羊颌下有毛髯，少数母羊部分有毛髯。颈长短适中。公羊雄性特征明显，睾丸发育良好。	35	30
体躯	前胸深广，肋骨开张，背腰平直。尻部略斜，肌肉发达。	35	35
乳房	乳房发育良好，乳头大小匀称。		10
四肢及蹄	四肢粗壮端正，蹄质坚实。	15	10
被毛	全身被毛黑色，有光泽。乐至型少数头顶部有"栀子花"样白毛	15	15
合计		100	100

体型外貌等级划分见表2。

表2 体型外貌等级

等 级	公 羊	母 羊
特	≥95	≥95
一	≥90	≥85
二	≥85	≥80
三	≥80	≥75

3.2 体重体尺等级划分

体重体尺等级划分见表3。

表3 体重体尺等级划分

年龄	类型与等级		公羊				母羊			
			体重(kg)	体高(cm)	体长(cm)	胸围(cm)	体重(kg)	体高(cm)	体长(cm)	胸围(cm)
六月	金堂型	特	35	63	65	73	30	60	62	68
		一	31	61	63	69	27	58	60	65
		二	28	59	61	66	25	56	58	63
		三	25	57	59	64	23	54	56	60
	乐至型	特	33	62	65	72	30	59	61	67
		一	30	60	63	69	26	57	59	64
		二	27	58	61	66	24	55	57	61
		三	24	56	59	64	22	53	55	57
周岁	金堂型	特	46	66	72	80	39	60	65	72
		一	42	64	70	78	36	59	63	70
		二	38	62	68	76	33	57	61	68
		三	34	60	66	74	30	55	59	66
	乐至型	特	45	65	72	78	39	59	64	71
		一	41	63	70	76	35	57	62	69
		二	37	61	68	74	32	55	60	67

年龄	类型与等级		公羊				母羊			
			体重(kg)	体高(cm)	体长(cm)	胸围(cm)	体重(kg)	体高(cm)	体长(cm)	胸围(cm)
		三	33	59	66	72	29	53	58	65
成年	金堂型	特	72	80	90	100	55	70	76	88
		一	65	75	86	96	50	65	70	84
		二	60	70	82	92	45	62	65	80
		三	55	65	78	88	40	59	60	76
	乐至型	特	76	81	92	102	53	68	77	89
		一	68	76	87	98	48	64	73	85
		二	63	71	83	94	43	61	69	81
		三	58	66	79	90	38	58	65	77

注：成年公羊3岁，成年母羊2.5岁

3.3 繁殖性能等级划分

3.3.1 母羊繁殖性能

母羊繁殖性能等级划分见表4。

表4 母羊繁殖性能等级划分

项 目	特	一	二	三
年产胎次(胎)	≥2.0	≥1.70	≥1.5	≥1.3
胎产羔数(只)	≥2.5	≥2.4	≥2.2	≥2.0

3.3.2 种公羊精液品质

种公羊每次射精量1.0ml以上,精子密度每毫升达20亿以上，活力0.7以上。

3.4 个体品质等级评定

3.4.1 母羊

母羊个体品质按照体重体尺、繁殖性能、体型外貌经济重要性权重进行评定等级，其中体重体尺占0.6、繁殖性能占0.3、体型外貌占0.1，详见表5。

表5 母羊个体品质等级评定

体型外貌	体 重 体 尺															
	特				一				二				三			
	繁殖性能				繁殖性能				繁殖性能				繁殖性能			
	特	一	二	三	特	一	二	三	特	一	二	三	特	一	二	三
特	特	特	特	一	一	一	一	二	一	二	二	二	二	二	三	三
一	特	特	一	二	一	一	二	二	二	二	二	二	二	三	三	三
二	特	一	二	二	一	二	二	二	二	二	二	三	三	三	三	三
三	一	一	二	二	二	二	三	三	二	二	三	三	三	三	三	三

3.4.2 公羊

公羊个体品质按照体重体尺、体型外貌经济重要性权重进行评定等级，其中体重体尺占0.65、体型外貌占0.35，详见表6。

表6 公羊个体品质等级评定

体型外貌	体　重　体　尺			
	特	一	二	三
特	特	一	二	三
一	特	一	二	三
二	一	一	二	三
三	二	二	二	三
