# Supplementary figures and images for: Whole-genome sequencing identified candidate genes associated with high and low litter size in Chuanzhong black goats
Source: Front Vet Sci. 2024 Sep 20;11:1420164. doi: 10.3389/fvets.2024.1420164 (PMC11449896; doi:10.3389/fvets.2024.1420164)

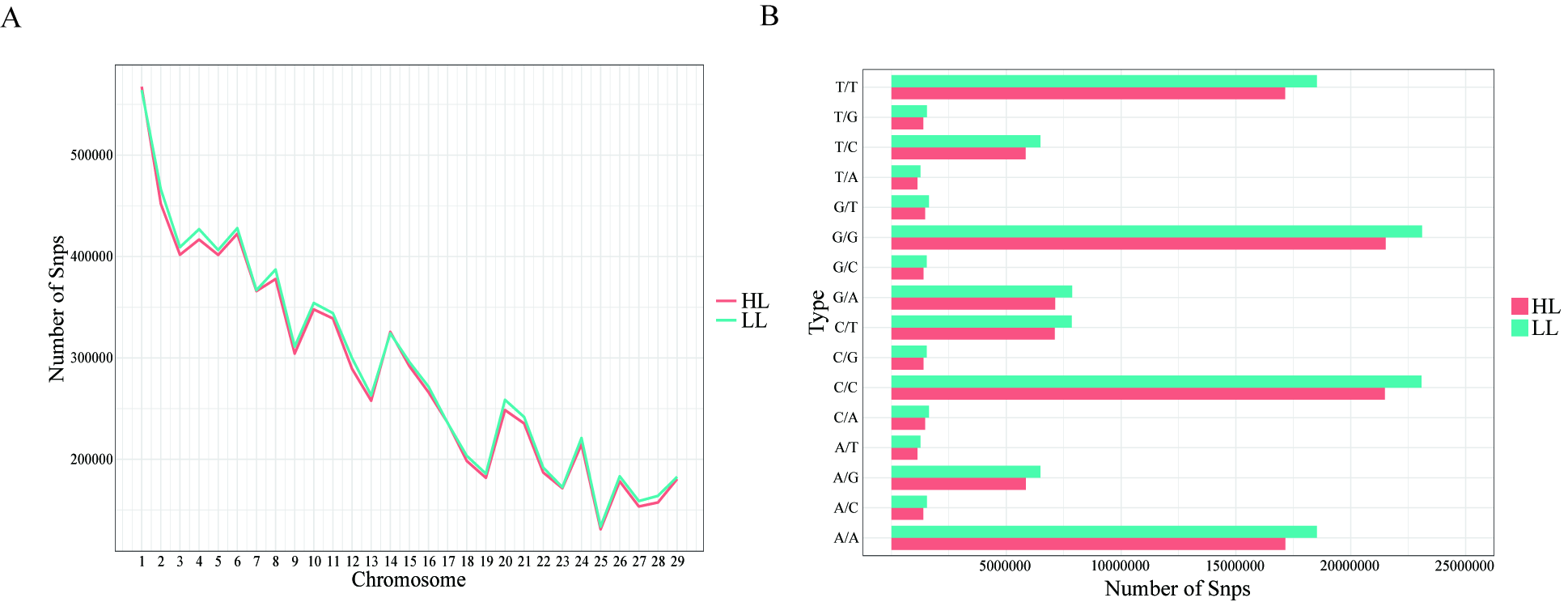

Supplement: Supplementary file 3 [file Image_1.TIF]
