# Supplementary material for: Whole-genome sequencing identified candidate genes associated with high and low litter size in Chuanzhong black goats
Source: Front Vet Sci. 2024 Sep 20;11:1420164. doi: 10.3389/fvets.2024.1420164 (PMC11449896; doi:10.3389/fvets.2024.1420164)
Supplement: Supplementary file 4 [file Table_1.DOCX]

Table S1 Summary of sequencing data quality status

| sample | Raw data | Effective data | Effective data rate | Average base error rate | Q20(%)^1^ | Q30(%)^2^ | GC content (%)^3^ |
| --- | --- | --- | --- | --- | --- | --- | --- |
| H1 | 27,843,832,200 | 27,649,102,800 | 99.30 | 0.03 | 97.64 | 93.34 | 43.72 |
| H2 | 30,256,776,900 | 29,683,815,300 | 98.11 | 0.03 | 96.91 | 91.86 | 47.05 |
| H3 | 29,662,381,500 | 29,443,012,200 | 99.26 | 0.03 | 97.37 | 92.39 | 43.55 |
| H4 | 27,814,504,200 | 27,618,588,900 | 99.30 | 0.03 | 97.34 | 92.65 | 43.62 |
| H5 | 28,073,487,000 | 27,882,138,300 | 99.32 | 0.03 | 97.55 | 93.10 | 43.30 |
| H6 | 26,578,760,100 | 26,406,888,300 | 99.35 | 0.03 | 97.44 | 92.85 | 43.27 |
| H7 | 30,786,686,700 | 30,546,573,900 | 99.22 | 0.03 | 97.16 | 92.28 | 44.21 |
| H8 | 34,228,209,600 | 33,967,403,700 | 99.24 | 0.03 | 97.42 | 92.84 | 43.54 |
| H9 | 29,552,629,200 | 29,336,811,600 | 99.27 | 0.03 | 97.53 | 92.97 | 43.06 |
| H10 | 28,615,713,000 | 28,427,135,400 | 99.34 | 0.03 | 96.86 | 91.53 | 43.52 |
| H11 | 30,323,067,600 | 29,927,976,600 | 98.70 | 0.03 | 97.23 | 92.77 | 46.14 |
| H12 | 32,162,967,000 | 31,930,486,500 | 99.28 | 0.03 | 96.74 | 91.24 | 43.05 |
| H13 | 29,870,390,100 | 29,705,223,900 | 99.45 | 0.03 | 97.05 | 91.69 | 42.67 |
| H14 | 30,049,663,200 | 29,826,423,900 | 99.26 | 0.03 | 97.29 | 92.34 | 43.27 |
| H15 | 27,483,879,000 | 27,272,088,600 | 99.23 | 0.03 | 97.34 | 92.62 | 42.73 |
| L1 | 28,270,083,600 | 27,854,524,800 | 98.53 | 0.03 | 96.83 | 91.94 | 46.79 |
| L2 | 30,463,497,900 | 30,244,026,000 | 99.28 | 0.03 | 97.19 | 92.12 | 43.36 |
| L3 | 32,409,471,900 | 32,156,407,800 | 99.22 | 0.03 | 97.48 | 92.96 | 43.20 |
| L4 | 28,160,716,800 | 27,989,217,600 | 99.39 | 0.03 | 97.20 | 92.17 | 43.16 |
| L5 | 27,703,572,000 | 27,501,890,100 | 99.27 | 0.03 | 97.38 | 92.67 | 42.15 |
| L6 | 28,712,619,600 | 28,515,085,500 | 99.31 | 0.03 | 97.44 | 92.83 | 41.97 |
| L7 | 27,628,634,100 | 27,444,917,100 | 99.34 | 0.03 | 97.50 | 92.97 | 42.48 |
| L8 | 27,288,080,700 | 27,118,727,100 | 99.38 | 0.03 | 97.25 | 92.40 | 42.54 |
| L9 | 29,785,053,300 | 29,587,171,500 | 99.34 | 0.03 | 97.26 | 92.38 | 42.02 |
| L10 | 28,735,499,100 | 28,576,833,000 | 99.45 | 0.03 | 97.39 | 92.67 | 41.86 |
| L11 | 26,732,475,300 | 26,567,756,400 | 99.38 | 0.03 | 97.62 | 93.23 | 41.90 |
| L12 | 30,159,567,600 | 29,980,689,900 | 99.41 | 0.03 | 97.34 | 92.57 | 41.73 |
| L13 | 26,976,734,700 | 26,800,857,300 | 99.35 | 0.03 | 97.36 | 92.31 | 41.89 |
| L14 | 26,850,299,400 | 26,678,291,100 | 99.36 | 0.03 | 97.40 | 92.72 | 42.22 |

Note: ^1^Q20: Percentage of bases with a quality value of 20 or more (with an error rate of 1% or less); ^2^Q30: Percentage of bases with a quality value of 30 or more (with an error rate of 0.1% or less); ^3^GC content: Percentage of bases G and C.
