# Supplementary material for: Whole-genome sequencing identified candidate genes associated with high and low litter size in Chuanzhong black goats
Source: Front Vet Sci. 2024 Sep 20;11:1420164. doi: 10.3389/fvets.2024.1420164 (PMC11449896; doi:10.3389/fvets.2024.1420164)
Supplement: Supplementary file 5 [file Table_2.DOCX]

Table S2 Sequencing depth and coverage statistics

| Sample | clean read | mapped_reads^1^ | mappingrate^2^ | Average depth^3^ | Coverage_1X^4^ | Coverage_4X^5^ |
| --- | --- | --- | --- | --- | --- | --- |
| H1 | 184327352 | 183741747 | 99.68% | 7.97 | 94.40% | 84.87% |
| H2 | 197892102 | 197070918 | 99.59% | 6.82 | 93.73% | 70.53% |
| H3 | 196286748 | 195828549 | 99.77% | 7.54 | 94.43% | 83.97% |
| H4 | 184123926 | 183646427 | 99.74% | 8.06 | 94.57% | 86.20% |
| H5 | 185880922 | 185463864 | 99.78% | 7.96 | 94.47% | 85.87% |
| H6 | 176045922 | 175580490 | 99.74% | 7.76 | 94.45% | 85.30% |
| H7 | 203643826 | 203000827 | 99.68% | 8.46 | 94.74% | 86.92% |
| H8 | 226449358 | 225753656 | 99.69% | 9.61 | 94.87% | 90.21% |
| H9 | 195578744 | 195141872 | 99.78% | 8.48 | 94.62% | 87.84% |
| H10 | 189514236 | 188851924 | 99.65% | 8.02 | 94.70% | 86.66% |
| H11 | 199519844 | 198767130 | 99.62% | 8.6 | 94.63% | 83.35% |
| H12 | 212869910 | 212194400 | 99.68% | 8.59 | 94.69% | 88.67% |
| H13 | 198034826 | 197363850 | 99.66% | 8.36 | 94.61% | 88.05% |
| H14 | 198842826 | 198299421 | 99.73% | 8.49 | 94.62% | 88.31% |
| H15 | 181813924 | 181330545 | 99.73% | 7.81 | 94.37% | 85.48% |
| L1 | 185696832 | 184831588 | 99.53% | 7.67 | 94.18% | 76.99% |
| L2 | 201626840 | 201074473 | 99.73% | 8.35 | 94.73% | 87.90% |
| L3 | 214376052 | 213857701 | 99.76% | 9.12 | 94.66% | 88.99% |
| L4 | 186594784 | 186133662 | 99.75% | 7.84 | 94.62% | 86.45% |
| L5 | 183345934 | 182882932 | 99.75% | 7.82 | 94.38% | 85.56% |
| L6 | 190100570 | 189583556 | 99.73% | 8.2 | 94.45% | 86.24% |
| L7 | 182966114 | 182551873 | 99.77% | 7.94 | 94.35% | 85.92% |
| L8 | 180791514 | 180296904 | 99.73% | 7.86 | 94.42% | 85.83% |
| L9 | 197247810 | 196747549 | 99.75% | 8.33 | 94.54% | 87.24% |
| L10 | 190512220 | 190067359 | 99.77% | 8.16 | 94.43% | 86.39% |
| L11 | 177118376 | 176716673 | 99.77% | 7.7 | 94.36% | 84.55% |
| L12 | 199871266 | 199406452 | 99.77% | 8.38 | 94.41% | 86.75% |
| L13 | 178672382 | 178212592 | 99.74% | 7.21 | 94.18% | 82.17% |
| L14 | 177855274 | 177387438 | 99.74% | 7.55 | 94.32% | 84.57% |

Note: ^1^Mapped reads: the number of reads aligned to the reference (including single-end alignment and double-end alignment); ^2^Mapping rate: Mapping rate, the number of reads matched to the reference genome divided by the number of reads of valid sequencing data; ^3^Average depth: average sequencing depth, the total number of bases aligned to the reference genome divided by the genome size; ^4^Coverage at least 1X: percentage of the genome with loci covered by at least 1 base in the reference genome; ^5^Coverage at least 4X: percentage of the genome with loci covered by at least 4 bases in the reference genome.
